# Formation of stable and responsive collective states in suspensions of active colloids

**DOI:** 10.1038/s41467-020-16161-4

**Published:** 2020-05-21

**Authors:** Tobias Bäuerle, Robert C. Löffler, Clemens Bechinger

**Affiliations:** Fachbereich Physik, Universität Konstanz, Konstanz, D-78464 Germany

**Keywords:** Engineering, Phase transitions and critical phenomena

## Abstract

Many animal species organise into disordered swarms, polarised flocks or swirls to protect from predators or optimise foraging. Previous studies suggest that such collective states are related to a critical point, which could explain their balance between robustness to noise and high responsiveness regarding external perturbations. Here we provide experimental evidence for this idea by investigating the stability of swirls formed by light-responsive active colloids which adjust their individual motion to positions and orientations of neighbours. Because their behaviour can be precisely tuned, controlled changes between different collective states can be achieved. During the transition between stable swirls and swarms we observe a maximum of the group’s susceptibility indicating the vicinity of a critical point. Our results support the idea of system-independent organisation principles of collective states and provide useful strategies for the realisation of responsive yet stable ensembles in microrobotic systems.

## Abstract (web summary)

Living organisms, like fish and bacteria, frequently change their pattern as a group to cope with environment. Here, Bäuerle et al. reproduce this phenomenon using a synthetic system of controllably interactive colloids to show their collective motions that indicates being close to a critical point.

## Introduction

Living organisms frequently arrange into spatio-temporal patterns being classified as disordered swarms, polarised flocks or rotating groups (also termed swirls or vortices)^1–5^. Because such states are observed in many animal species, including fish^6,7^, birds^8^, insects^9^ and down to bacteria^10^, this suggests the presence of stable and size-independent overarching organisation principles. In order to cope with noise and external perturbations, collective states should keep a balance between robustness and flexibility regarding changing environmental conditions. Such conflicting needs may be resolved by collective states being close to a critical point^11,12^. Indeed, the analysis of velocity fluctuations in starling flocks^8,13^ and swarms of midges^14,15^ reveals evidence of critical behaviour, e.g., the existence of correlation lengths largely exceeding the interaction range between individuals. Further evidence of a close relation between collective and critical states is obtained from theoretical studies of interacting particles that adjust their motion to their neighbours via local interaction rules^16–20^. For example, such simulations demonstrate a maximum of the responsiveness to perturbations in groups of fish at the transition from milling to schooling, which strongly resembles the behaviour at a critical phase transition^16^. Opposed to simulations that typically consider ideal particles with monodisperse properties and simplified interactions^21,22^, experimental observations are scarce. In addition, interaction rules in living systems are frequently only poorly understood, and it remains unclear, how systematic variations of their collective behaviour can be achieved. Therefore, despite its implications, the idea of a possible link between criticality and collectivity requires further tests under experimental conditions. Here we study the collective properties of self-propelling, i.e., active particles (APs) that have been demonstrated to closely resemble the behaviour of living microorganisms^23–27^. Opposed to the majority of studies dealing with APs whose propulsive properties are typically controlled at the group level^28–36^, there has been recent progress in controlling the AP motility on a single-particle level in response to neighbours using optical feedback loops^37–39^. Here, we go one step further by controlling not only the magnitude but also the direction of the particle propulsion, which is central for our work since it allows to add alignment interactions between APs. The AP interaction rules in our experiments are inspired by social interactions of living organisms, which are often described by a combination of avoidance, alignment or attraction to neighbours^21,22,40–43^. For the specific interaction rules applied here, we observe the spontaneous formation of swirls, i.e., rotating groups of APs that are either very robust or highly responsive to fluctuations, depending on small changes of interaction parameters. In particular, we observe a continuous transition between swirls and swarms, which is accompanied by large fluctuations resembling critical behaviour.

## Results

### Experimental realisation

APs that respond to positions and orientations of neighbours are fabricated from silica particles with diameter *σ* = 6.3 μm, and coated with a 80-nm carbon cap on one side. They are suspended in a binary fluid mixture near room temperature and contained in a thin sample cell. When a laser spot is directed to an AP, it self-propels with velocity ${\hat{\mathbf{v}}} = v{\hat{\mathbf{u}}}$ with the cap at the back, where ${\hat{\mathbf{u}}}$ is a unit vector along the particle orientation^37,38^ (“Methods”). The magnitude of the velocity depends on the illuminating intensity, and was kept constant to yield *ν* = 0.5 μm s^−1^. The swimming direction of APs is controlled by a small offset of the illuminating laser spot relative to the AP centre (otherwise the direction is randomised due to rotational diffusion). This causes an intensity gradient across the AP, which leads to its reorientation^44,45^, with an angular turning velocity of ≈4° s^−1^ (“Methods”). Individual particle steering is achieved by a feedback loop, which controls the orientation of each particle based on the evaluation of the positions and orientations of all particles. This information is acquired with a rate of 5 Hz by optical video microscopy and subsequent image evaluation. With this information, we are able to compute the visual perception of each particle in real time, and to adjust its swimming direction independently (“Methods”). Unless otherwise stated, experiments were carried out with about 50 APs each.

**Fig. 1:**
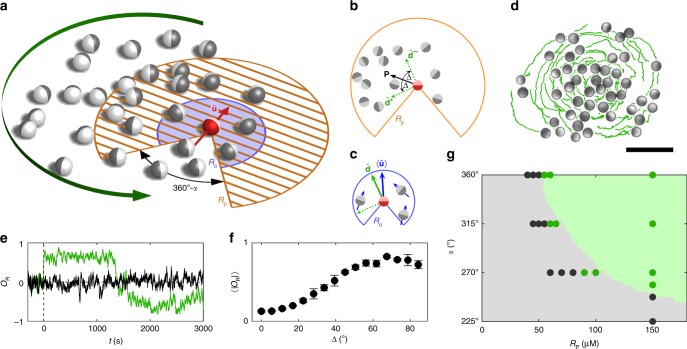
Swirl formation of socially interacting active particles. **a** Illustration of interaction rule leading to circular group motion. An AP (red) senses the positions and orientations of neighbours within its field of vision with angle *α* and range *R*_p_ (orange dashed region and **b**) and *R*_o_ (blue region and **c**), respectively. **b** After having determined the direction **P** to the centre of mass of sensed particles within distance *R*_p_, the AP selects one of two possible swimming directions ${\hat{\mathbf{d}}}^ +$ or ${\hat{\mathbf{d}}}^ -$ (green arrows) that deviate by the angle Δ to the left or the right relative to **P**. **c** The selection of the swimming direction ${\hat{\mathbf{d}}}$ depends on which of the two possibilities is closer to the mean orientation $\left\langle {{\hat{\mathbf{u}}}} \right\rangle$ of neighbours being closer than *R*_o_. **d** Experimental snapshot of a counterclockwise rotating swirl for *α* = 360°, Δ = 67.5° and *R*_p_ = ∞. The solid lines show trajectories of 150 s. Scale bar is 30 μm. **e** Time evolution of the rotational order parameter *O*_R_ for Δ = 67.5° (green) and Δ = 0 (black). **f** $\left\langle {\left| {O_{\mathrm{R}}} \right|} \right\rangle$ as a function of the deviation angle Δ. Error bars correspond to the standard deviation of different experiments. **g** Existence range of cohesive swirls (green) as a function of *α* and *R*_p_ determined from experiments (symbols) and numerical simulations (coloured areas) for Δ = 67.5°.

In our experiments, APs perform directional changes in response to their neighbours within their field of vision, the latter characterised by a viewing angle *α* and range *R*. First, each AP (particle index *i*) determines the current direction **P**_*i*_ to the centre of mass of all particles within *α* and the position detection range *R*_p_ (orange dashed region in Fig. 1a, b). Then it sets its swimming direction towards **P**_*i*_, but with a constant angular deviation Δ to the left (+) or the right (−). From the two possible swimming directions ${\hat{\mathbf{d}}}_i^ \pm$ (Fig. 1b), the particle chooses the one that minimises the difference relative to the mean orientation of close neighbours $\left\langle {{\hat{\mathbf{u}}}_i} \right\rangle$, the latter sensed within angle *α* and the orientation detection range *R*_o_ (blue area in Fig. 1a, c). Contrary to most zonal models where sensing regions of attraction and orientation are spatially separated^21,40,41,46^, here they overlap (Fig. 1a). Throughout this work, we set *R*_o_ = 25 μm ≈ 4*σ*. To hinder particle collisions, swimming directions of APs are reversed (as in ref. ^41^) when their clearance is below 0.25*σ*.

Figure 1d shows a snapshot of a swirl of 50 APs (Supplementary Movie 1). To quantify its rotational motion, we use the rotational order parameter $O_{\mathrm{R}} = \frac{1}{N}\mathop {\sum}\nolimits_{i = 1}^N {\left( {{\hat{\mathbf{r}}}_i \times {\hat{\mathbf{u}}}_i} \right) \cdot {\mathbf{e}}_z}$, which measures the in-plane circular motion of the group (Fig. 1e). Here, ${\hat{\mathbf{r}}}_i$ denotes the unit vector of the AP position relative to the total centre of the vortex and **e**_*z*_ the unit vector perpendicular to the sample plane. Apart from a sudden change of sign of *O*_R_, i.e., a change in the sense of rotation, |*O*_R_| remains at about ≈0.8, confirming a high degree of collective circular group motion.

The collective properties strongly depend on the interaction parameters. This is seen in Fig. 1f where we show the dependence of $\left\langle {\left| {O_{\mathrm{R}}} \right|} \right\rangle$ on the deviation angle Δ. At low Δ, particles propel more or less directly towards the group centre where they form a cohesive disordered swarm lacking angular motion (black line in Fig. 1e). With increasing Δ, the angular motion of APs becomes more and more pronounced, which leads to an increase of $\left\langle {\left| {O_{\mathrm{R}}} \right|} \right\rangle$. With increasing Δ, however, also the spatial extent of the swirl increases (Supplementary Fig. 1). This leads to fewer next neighbours within *R*_o_, eventually leading to a decrease of $\left\langle {\left| {O_{\mathrm{R}}} \right|} \right\rangle$ above Δ ≈ 70°. For Δ = 90°, cohesion of the group is entirely lost, and swirls are no longer observed. Swirl formation is also affected by the choice of the vision angle *α* and the position detection range *R*_p_. In general, we obtain stable swirls at large values of *α* and *R*_p_ (Fig. 1g, “Methods”). Otherwise, the number of sensed neighbours around each AP becomes too small, which leads to the loss of cooperative motion resulting in fragmentation of swirls (Supplementary Movie 2).

To corroborate our experimental observations, we also performed numerical simulations using a minimal model based on an overdamped Langevin equation (“Methods”). Even though such models provide only a crude approximation of the rather complex interactions in our system, the stability range is in fair agreement with our experimental data. Because the thermal noise can be easily varied in simulations, we also investigated the impact of noise on swirl formation. As expected, $\left\langle {\left| {O_{\mathrm{R}}} \right|} \right\rangle$ decreases with increasing noise strength (Supplementary Fig. 2). Comparison of our interaction rule with standard zonal models, however, reveals a much weaker degrading effect of noise on $\left\langle {\left| {O_{\mathrm{R}}} \right|} \right\rangle$. This difference results from the fact that, in standard zonal models, the velocity direction of each individual is a superposition of the motion towards the centre-of-mass **P** and the mean orientation of the next neighbour $\left\langle {{\hat{\mathbf{u}}}} \right\rangle$. Because of the fixed deviation angle in our interaction rule, small variations of $\left\langle {{\hat{\mathbf{u}}}} \right\rangle$ have a smaller influence on the cooperative behaviour that renders our swirls rather robust against orientational disorder, sensorial errors or number fluctuations of neighbouring particles.

### Swirl dynamics

**Fig. 2:**
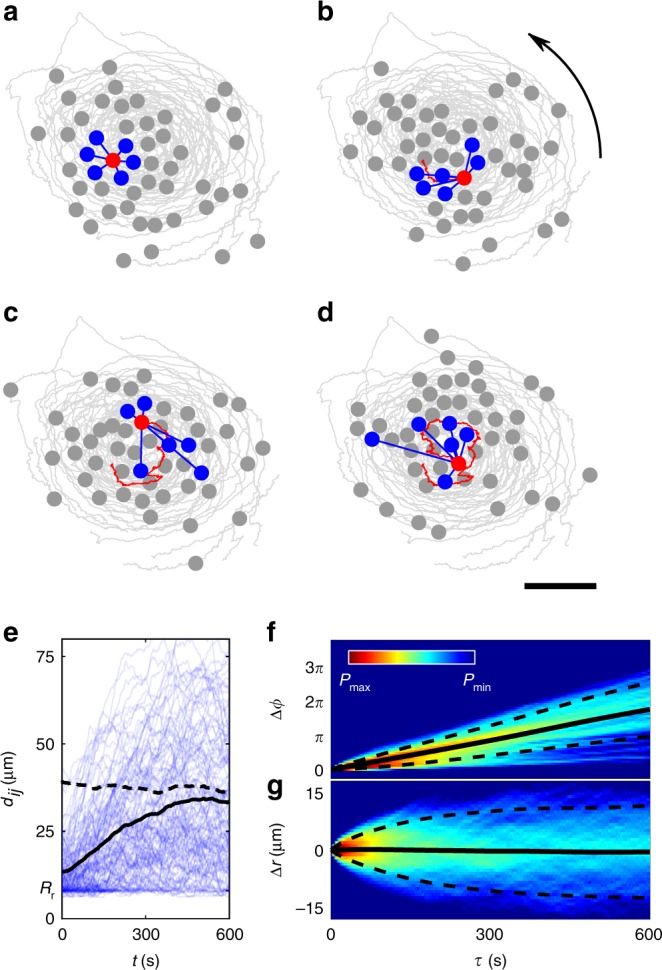
Temporal decay of next-neighbour correlations. **a**–**d** Snapshots of the positions of a single AP (red) and its original (*t* = 0 s) nearest neighbours (blue) at times *t* = [0, 100, 300 and 600] s. Grey lines correspond to trajectories over 600 s each. The scale bar is 30 μm. **e** Time dependence of all distances to original (*t* = 0 s) next neighbours (blue lines). The mean value of these curves (solid line) converges to the average of all possible pair distances (dashed line). The lower limit *R*_r_ indicates the repulsion range between APs. **f**, **g** Time-dependent probability distributions of the angular and radial AP displacements (both measured relative to the swirl centre). From the slope in **c**, one obtains a swirl’s revolution time of about 600 s. Dashed lines show the standard deviation. All data are obtained for *α* = 360°, Δ = 67.5°, *R*_p_ = ∞.

To maintain an ideal local order within a swirl, all group members should travel on concentric orbits with constant angular velocity. Such conditions, however, are not met in our experiments. In fact, APs rapidly change their positions relative to their neighbours (Fig. 2a–d). Within about 600 s (approx. one swirl rotation), initial neighbours distribute across the swirl, some of them even propelling in opposite directions. The rapid loss of neighbours is also seen in Fig. 2e where we plot how the distance *d*_*ij*_ of all APs *i* to their original (*t* = 0) next neighbour *j* changes over time. These separations rapidly randomise owing to the combination of thermal noise, particle collisions and variations in particle velocities. Interestingly, the mean value of $\langle {d_{ij}\left( t \right)} \rangle$ (where the bracket corresponds to averaging over all particles *i* and *j*) shown as solid black line rapidly converges to the mean value of all particle distances (dashed line). This demonstrates that stable swirls can exist even when nearest neighbours are rapidly lost. We also measured the time dependence of the probability distributions of the angular and radial AP displacements *P*(ΔΦ) (Fig. 2f) and *P*(Δr) (Fig. 2g), both values defined relative to the swirl centre. Even though the distributions are rather broad, the mean value of *P*(ΔΦ) (solid line) shows an almost linear increase that suggests an overall uniform swirl rotation.

**Fig. 3:**
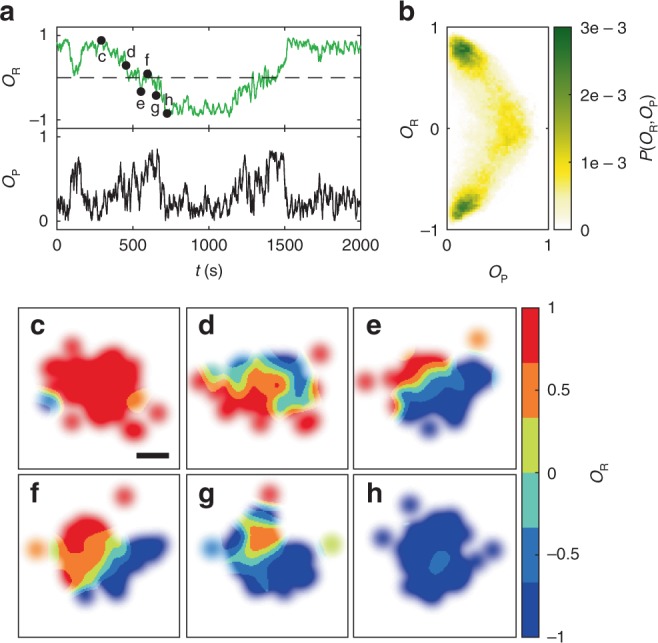
Spontaneous reversal of rotation direction. **a** Time-dependent rotational and orientational order parameters *O*_R_ and *O*_P_ during a spontaneous change of the swirl’s sense of rotation. **b** Histogram of the correlation between *O*_R_ and *O*_P_ obtained from measurements, including about 50 rotation reversals. **c**–**h** Spatially resolved rotational order parameter at different times (marked in **a**). Scale bar is 30 μm. All measurements were performed for *α* = 360°, *R*_p_ = ∞, Δ = 67.5°.

Despite random changes in the sense of rotation, swirls remain remarkably stable. This is shown in Fig. 3a where we compare rotational (*O*_R_) and orientational order during a spontaneous rotation reversal (Δ = 67.5°). Orientational order (polarisation) is characterised by the corresponding order parameter $O_{\mathrm{P}} = \frac{1}{N}\left| {\mathop {\sum}\nolimits_{i = 1}^N {{\hat{\mathbf{u}}}_i} } \right|$. Both quantities are strongly anti-correlated, i.e., *O*_P_ becomes largest when *O*_R_ = 0. This indicates that local order is not entirely lost during a rotation reversal of the group. The colour-coded probability distribution of the correlation between *O*_P_ and *O*_R_ demonstrates that the group stays most of the time within a swirling state, and that directional changes of the swirl’s rotation are accompanied by a metastable flock with high polarisation (Fig. 3b). To understand how rotational order changes within a swirl during a rotation reversal, we calculated the spatially resolved rotational order as a function of time (Fig. 3c–h, “Methods”). Typically, rotation reversals start from the edges, where particles have fewer neighbours and are therefore more affected by fluctuations of their environment (d, blue region). When *O*_R_ ≈ 0, the group comprises of two subregions with opposite sense of rotation (e, f). This metastable intermediate state has a high polarisation and rapidly decays back into a swirl (g, h). As a side remark, we mention that stable flocks with high polarisation *O*_P_ can be obtained within our interaction model when *R*_o_ becomes sufficiently large (Supplementary Fig. 3).

### Critical behaviour

**Fig. 4:**
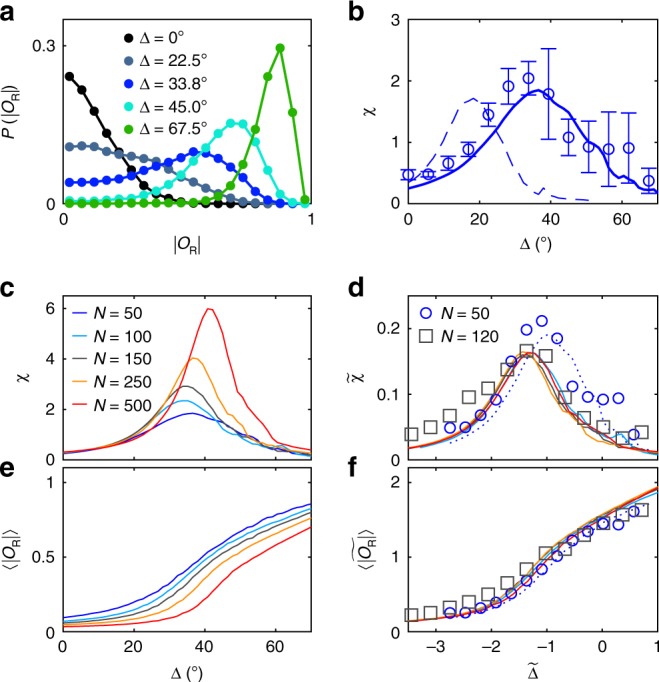
Susceptibility and finite-size scaling. **a** Experimental probability distribution of the rotational order parameter |*O*_R_| for different values of Δ showing a continuous transition between a swarm (black) and a swirl (green) of a group of *N* = 50 APs. **b** Corresponding susceptibility *χ* as a function of the deviation angle Δ (symbols). Error bars correspond to the standard deviation obtained from different measurements. Lines correspond to simulations where *D*_R,sim_ is identical (dashed) and three times larger (solid) compared with the experimental value *D*_R,exp_ = 0.0028 s^−1^. **c** *χ* vs. Δ obtained from simulations for different particle numbers *N* = [50, 100, 150, 250, 500] and *D*_R,sim_ = 3*D*_R,exp_. **d** Corresponding rescaled susceptibility $\widetilde \chi = \chi \cdot N^{ - \gamma /\nu }$ vs. the rescaled deviation angle $\widetilde {\mathrm{\Delta}} = \frac{{\Delta} \, - \, {\Delta}_{c}}{{\Delta}_{c}} \cdot N^{1/\nu}$. The critical exponents *ν*, *γ* and the value of Δ_c_ were used as free fitting parameters. The best data collapse is achieved for *ν* = 3.87, *γ* = 2.24 and Δ_c_ = 56.15°. **e** $\left\langle {\left| {O_{\mathrm{R}}} \right|} \right\rangle$ vs. Δ for simulations with *N* = [50, 100, 150, 250, 500]. **f** Corresponding rescaled order parameter $\widetilde {\left\langle {\left| {O_{\mathrm{R}}} \right|} \right\rangle } = \left\langle {\left| {O_{\mathrm{R}}} \right|} \right\rangle \cdot N^{\beta /\nu }$ vs. ${\tilde{\mathrm{\Delta }}}$ with *β* as a free fitting parameter (*ν* and Δ_c_ were taken from **d**). Best collapse is achieved for *β* = 0.66. Symbols in **d** and **f** show experimental data for *N* = 50 and *N* = 120.

In Fig. 4a, we show how the probability distribution of |*O*_R_| varies upon changing the deviation angle Δ. For Δ = 67.5°, the distribution exhibits a single peak around |*O*_R_| ≈0.8 and a small probability at |*O*_R_| = 0, in agreement with the above observation of stable swirls with rare rotation reversals under such conditions (Fig. 1e). Decreasing Δ leads to a continuous increase of the probability around |*O*_R_| = 0 at the expense of the peak at large |*O*_R_|, which becomes smaller but also broader. For Δ = 22.5° and below, a single broad peak at |*O*_R_| = 0 is observed. When calculating the susceptibility *χ* of the group (“Methods”), this leads to a peak around Δ = 33.75° (Fig. 4b). When comparing these results with *χ* obtained from numerical simulations, we find also a maximum, however, with the peak shifted to much smaller deviation angles (dashed line). This difference is attributed to the fact that the simulations do not fully capture all experimental details. For example, collisions between APs typically affect their orientation, opposed to the ideal collision process assumed in simulations. To account for this effect, and to obtain best agreement with the experimental data in Fig. 4b, we have increased the rotational diffusion constant *D*_R_ in the simulations to three times the experimental value of an isolated AP (solid line). In combination with the continuous change of the order parameter distribution (Fig. 4a), a maximum of the susceptibility as a function of an external control parameter suggests the occurrence of a critical phase transition. To further strengthen this assumption, we have performed a finite-size scaling that predicts the general size dependence of critical phenomena (see e.g. ref. ^47^). Therefore, we have varied the particle number *N* in our simulations. The corresponding susceptibility is shown in Fig. 4c for 50 ≤ *N* ≤ 500. Clearly, the position and the width of the peak vary with *N*. After application of a finite-size scaling, the data (in particular those for *N* > 50) collapse on a single curve (Fig. 4d, “Methods”). When adding our experimental data for *N* = 50 and *N* = 120 (without any adjustable parameter), they show good agreement with the predicted behaviour. A similar finite-size scaling is applied to the orientational order parameter $\left\langle {\left| {O_{\mathrm{R}}} \right|} \right\rangle$ that also leads to a data collapse (Fig. 4e, f, “Methods”). The applicability of the finite-size scaling method to our data gives further evidence that the transition between swirls and swarm is indeed a critical phase transition.

### Response to perturbations

**Fig. 5:**
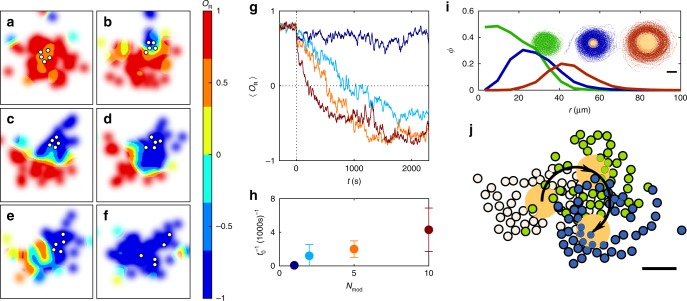
Stability of swirls against perturbations. **a**–**f** Spatially resolved *O*_R_ at times *t* = [0, 120, 240, 360, 480, 600] s for *N*_mod_ = 5 (positions shown as white circles). **g** Time dependence (averaged over four measurements each) of *O*_R_ after *N*_mod_ = [1, 2, 5, 10] (blue to red) APs with only clockwise motion are activated at *t* = 0 s in a counterclockwise rotating swirl. **h** Inverse time $t_0^{ - 1}$ until the order parameter reaches zero for the first time. Error bars correspond to the standard deviation of different experiments. **i** Radial profile of the area fraction Φ for a swirl without obstacle (green) and with virtual obstacles of 15-μm (blue) and 30-μm (red) radius. Inset: the corresponding particle trajectories with obstacles as orange discs. **j** Snapshots of APs milling around an obstacle (orange disc) with 15-μm radius and moving along a circular trajectory (arrow) with 30-μm radius and a velocity of 0.025 μm s^−1^. All data are obtained for *α* = 360°, Δ = 67.5°, *R*_p_ = ∞. Scale bars are 30 μm.

To test the stability of swirls regarding individual variations of the interaction rules within the group, we investigated the influence of a subset of misbehaving particles, i.e., APs having a modified response to their neighbours^48^: opposed to above, where the swimming direction of each AP can deviate either to the left or the right relative to **P** (depending on the orientation of nearest neighbours); APs with modified response misalign their motion only to the left, independent of the orientation of neighbours. As a result, these APs have a strong bias towards a clockwise circular motion. In our experiments, we first waited until the swirl’s rotation was counterclockwise (*O*_R_ > 0). Then we suddenly applied the modified response rule to a number of *N*_mod_ active particles. Figure 5a–f shows that the temporal change in the local rotational order parameter, after five (out of 50) APs with modified behaviour are initialised in the central region of the swirl (open circles). It should be noted that the experiments were performed at parameters where stable swirls are observed (*α* = 360°, Δ = 67.5°, *R*_p_ = ∞). Opposed to a spontaneous rotation reversal that usually exhibits strong fluctuations in *O*_R_ (Fig. 3b), here a qualitatively different behaviour is observed: the modified APs act as nucleation centres and firmly impose their sense of rotation (*O*_R_ < 0) first to their immediate surroundings but later to the entire swirl. Figure 5g shows how the rotational order within the swirl changes when 1 ≤ *N*_mod_ ≤ 10 APs have been activated at *t* = 0 s. Already, *N*_mod_ = 2 (out of *N* = 50) APs are sufficient to—at least temporarily—reverse the rotation sense of the swirl. For *N*_mod_ ≥ 5, a persistent clockwise rotation is achieved. With increasing *N*_mod_, the time required to reverse the sense of rotation decreases (Fig. 5h). Interestingly, in all cases, swirls remain intact. This is not self-evident since APs with modified response enhance local collisions, which in general lead to swirl destabilisation.

We also investigated how swirls respond to an obstacle, which is a realistic scenario in living but also microrobotic systems (“Methods”). Figure 5i shows how the radial density distribution of a group of APs changes in the presence of a static spherical obstacle in the centre of the swirl. As a result, a hole forms in the middle of the swirl. When the obstacle is translated along a circular path, we observe an asymmetric deformation of the swirling APs with an accumulation (depletion) at the front (rear) of the obstacle (“Methods”). Notably, the swirl remains stable and follows the motion of the obstacle (Fig. 5j; Supplementary Movie 3). This opens an effective method to control the centre-of-mass motion of an entire group of milling particles by inserting and translating a single obstacle inside the swirl.

## Discussion

Experiments with freely programmable active particles, as demonstrated here, provide an effective approach to understand the behaviour of collective states in the absence of centralised control. Opposed to numerical simulations that typically consider minimalistic models and idealised interactions, particle properties under real conditions are typically more complex. Hydrodynamic coupling^49–51^, positional and orientational detection errors or manufacturing-related variations of particle properties and even malfunctioning are just a few effects that are difficult to implement in simulations, but are relevant in experimental systems. Therefore, active systems with enhanced motion control will reduce the gap between theoretically conceived and reliably working algorithms leading to stable collective behaviour^52–54^. Apart from providing insights, how general physical concepts can be helpful to tackle the complexity of collective behaviour in biological systems, our experimental approach provides a strategy on how to achieve similar collective phenomena in fully autonomous AP systems. In fact, the particle response used in our interaction rule can in principle also be implemented, e.g., in catalytically driven APs where (i) short-ranged repulsive interactions arise due to steric interactions, (ii) long-ranged attraction towards the centre of mass can be accomplished by positive chemotaxis^55^ and (iii) alignment interactions can be realised by either hydrodynamic^51,56^ or diffusiophoretic^57,58^ interactions. When the weights of these contributions are properly adjusted by a suitable design of the shape, thickness and material of the catalytic cap, swirls or flocks as observed here can also be expected in fully autonomous systems.

## Methods

### Experimental system

As APs, we use silica spheres with diameter *σ* = 6.3 μm being coated with a 80-nm carbon cap on one hemisphere. Particles are suspended in a critical water–lutidine mixture that is kept at *T* = 28 °C being several degrees below the critical temperature of *T*_C_ ≈ 34.15 °C. The entire suspension is confined in a 200-μm-thick sample cell, where translational and rotational motion is restricted to two dimensions due to gravity and hydrodynamic effects^59^. The translational and rotational diffusion constants are determined to *D*_T_ = 0.014 μm^2^ s^−1^ and *D*_R_ = 0.0028 s^−1^. Under uniform light illumination of an AP with a laser spot, the strong absorption at the carbon coating heats this hemisphere above the critical temperature, which leads to local demixing of the solvent and thus self-propulsion. For the experimental conditions of this work, particles propel opposite to the orientation of the capped hemisphere and perform a persistent random walk^60^. When the position of the illuminating laser spot is shifted relative to the AP centre, the corresponding light and temperature gradient across the particle leads to an inhomogeneous demixing profile, causing a reorientation of the particle opposite to the light gradient ∇*I*^45^.

### Experimental control of the particle alignment

**Fig. 6:**
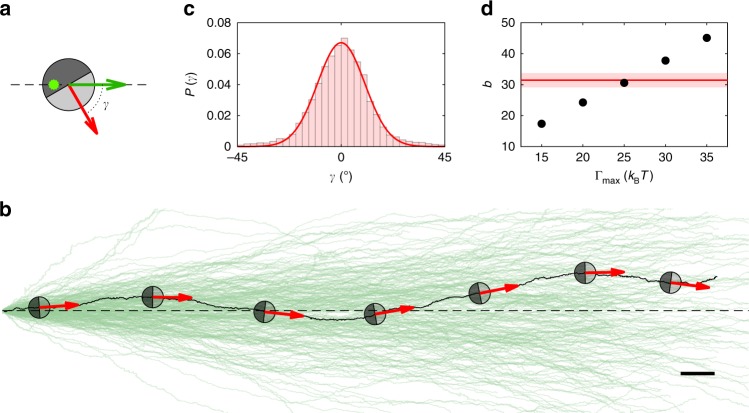
Calibration of AP reorientation. **a** Sketch of the experimental situation to measure active torques on an AP. The illuminating laser spot (green symbol) is displaced by 0.3 *σ* opposite to the steering direction (green arrow). This causes a torque on the particle, leading to alignment of the current particle orientation (red arrow) with the steering direction. **b** Measured particle trajectories (green) when APs are steered along a straight path to the right (dashed line). Scale bar is 10 μm. **c** Measured probability distribution of γ (coloured bars). In principle, γ can be measured by the optical contrast due to the light-absorbing carbon cap. This, however, leads to relatively large errors in γ. Therefore, γ has been obtained from the swimming direction averaged over 20 s. The solid line corresponds to a fit to *P*(γ) = *a* exp (*b*⋅cos(γ)). **d** Fit parameter *b* as obtained from simulated trajectories according to the same protocol as shown in **a**. To compare the experimental *P*(γ) with that obtained from numerical simulations, we first computed AP trajectories for different maximal torques Γ_max_. Following the identical evaluation procedure of γ as for the experimental data, this yields the parameter *b* which is shown here (symbols). Best agreement of the *b*-value of the experimental data (red line) is obtained for Γ_max_ = 25 *k*_B_*T*. The coloured band corresponds to the standard deviation of 6 independent experiments.

To control the alignment of APs independently, we take images of the particle configuration at a frequency of 5 Hz. The particle positions and orientations (defined as the vector from the capped side to the uncapped one) are obtained by real-time image analysis on a computer. This information is employed to direct a 532-nm laser beam to the particles with an acousto-optical deflector. Each particle is illuminated with a laser spot (beam waist *w* = 5 μm) for 8 μs every 2 ms. Because the remixing timescale of the water–lutidine mixture is about 100 ms, such a protocol ensures steady self-propulsion conditions. Throughout this work, the (time-averaged) intensity of the laser beam is *I*_0_ = 0.38 W mm^−2^, which leads to a propulsion velocity of *ν* = 0.5 μm s^−1^. The controlled reorientation of the APs towards the target direction ${\hat{\mathbf{d}}}$ is achieved by offsetting the laser spot 1.8 μm away from the particle centre opposite to the target direction ${\hat{\mathbf{d}}}$ (Fig. 6a) This results in a torque Γ_max_ ≈ 25 *k*_B_*T*, which corresponds to a rotation rate *ω*_max_ ≈ 4° s^−1^ (see also below).

### Characterisation of the orientational steering of APs

Under homogeneous particle illumination, APs self-propel with constant velocity and random changes of their swimming direction due to rotational diffusion. When subjected to a local light gradient ∇*I*, however, the AP’s mean orientation (i.e., swimming direction) becomes aligned opposite to ∇*I*^45^. This is due to the inhomogeneous heating of the carbon cap, which leads to an asymmetry of the fluid’s flow field around the AP relative to its orientation ${\hat{\mathbf{u}}}$. This results in a torque ${\mathrm{\Gamma }} \propto \nabla I \times {\hat{\mathbf{u}}}$ on the AP, which can be employed for steering APs towards a desired target direction ${\hat{\mathbf{d}}}$. To achieve temporal changes in ${\hat{\mathbf{d}}}$, the illumination gradient must be dynamically and independently adjusted for each particle. This is experimentally realised by the controlled displacement of the laser spot by 1.8 μm ≈ 0.3 *σ* away from the geometrical AP centre. To steer a particle along ${\hat{\mathbf{d}}}$ the displacement of the laser spot is chosen exactly opposite to ${\hat{\mathbf{d}}}$ (Fig. 6a). Then a local intensity gradient $\nabla I \propto - {\hat{\mathbf{d}}}$ is created as confirmed by integration of a Gaussian laser spot. This leads to a realigning torque Γ(*γ*) = −Γ_max_sin(*γ*), with *γ* the angle between ${\hat{\mathbf{d}}}$ and ${\hat{\mathbf{u}}}$ and Γ_max_ the maximum torque (${\hat{\mathbf{d}}} \bot {\hat{\mathbf{u}}}$). In analogy to an optical laser trap where the restoring forces vanish in the intensity maximum, the restoring torque acting on an AP becomes zero when ${\hat{\mathbf{d}}}$ and ${\hat{\mathbf{u}}}$ coincide, i.e., when the particle propels along the desired direction (orientational particle trapping). Due to rotational diffusion, however, deviations from perfect straight swimming occur. To quantify restoring torques, we measured the trajectory of APs with constant target direction to the right (Fig. 6b) over more than 200 μm, and determined the probability distribution *P*(*γ*) (Fig. 6c). Using the Boltzmann distribution, this can be written as $P\left( \gamma \right) \propto \exp \left( { - \frac{{V\left( \gamma \right)}}{{k_{\mathrm{B}}T}}} \right)$ with the effective aligning potential $V\left( \gamma \right) = - {\int} {{\mathrm{\Gamma }}\left( \gamma \right)d\gamma = - {\mathrm{\Gamma }}_{{\mathrm{max}}}\cos \left( \gamma \right)}$. Fitting the above expression to our data yields best agreement for Γ_max_ ≈ 25 *k*_B_*T* (Fig. 6d). Using the rotational mobility *μ* = *ω*/Γ and the Einstein relation, the largest reorientation rate is determined to $\omega _{{\mathrm{max}}} = \frac{{{\mathrm{\Gamma }}_{{\mathrm{max}}}}}{{k_{\mathrm{B}}T}}D_{\mathrm{R}} \approx 4^\circ \;{\mathrm{s}}^{ - 1}$.

### Quantification of swirl stability

To quantify stable (cohesive) swirls in the context of Fig. 1g, we have confirmed that the radius of gyration of the rotating group was changing by less than 10 μm.

### Calculation of local rotational order parameter

The spatially dependent order parameter in Figs. 3 and 5 is calculated according to $O_{\mathrm{R}}\left( {x,y} \right) = \frac{{\mathop {\sum}\nolimits_i {O_{R,i}\exp \left( { - \frac{{\left| {{\mathbf{r}}_i} \right|^2}}{{2\sigma ^2}}} \right)} }}{{\mathop {\sum}\nolimits_i {\exp \left( { - \frac{{\left| {{\mathbf{r}}_i} \right|^2}}{{2\sigma ^2}}} \right)} }}$ with the particle diameter *σ* = 6.3 μm and |**r**_*i*_| the distance between particle *i* and the position (*x*, *y*).

### Calculation of susceptibility

The susceptibility *χ* of a system is generally defined as $\chi = \frac{{\partial \left\langle O \right\rangle }}{{\partial h}}_{|h = 0}$, where $\left\langle O \right\rangle$ is the time- averaged mean of an order parameter *O* and *h* a weak external field. According to the fluctuation–dissipation theorem, *χ* is also related to the fluctuations of *O* in the stationary state, which leads to $\chi \propto N \cdot \left. {\left( {\left\langle O \right\rangle ^2 - \left\langle O \right\rangle ^2} \right.} \right)$, where *N* is the number of particles and angular brackets correspond to time-averaged quantities^16,61^. Because the rotational order parameter *O*_R_ changes between positive and negative values when the swirl changes its sense of rotation, its mean $\left\langle {O_{\mathrm{R}}} \right\rangle$ is zero, which leads to $\chi \propto N \cdot \left\langle {O_{\mathrm{R}}^2} \right\rangle$. This quantity, however, does not yield a maximum at the critical point, but increases with increasing rotational order, where *O*_R_ becomes the largest. This problem is well known also in the context of the magnetisation order parameter within the Ising model that is—in the absence of a magnetic field—also zero when averaged over different initial conditions. In such situations, the absolute value of the order parameter is used when calculating *χ* (see ref. ^61^). Accordingly, we use |*O*_R_|, rather than *O*_R_, when measuring the susceptibility.

For large Δ, there is a possibility for the group to enter a metastable, i.e., polarised state. When this happens (less than 20% of measurements), the measurement is interrupted as the group directly swims to the edge of our field of view, and therefore these measurements are neglected.

### Finite-size scaling

The correlation length in a critical system diverges when the system’s control parameter is changed towards the critical point. In simulations and our experiments, however, the system size is not infinite and thus limits the correlation length. This affects the relevant properties of the system, such as the order parameter and the susceptibility. Using the critical exponents of the system and the critical value of the control parameter, data for systems with different size can be rescaled to collapse onto one single curve^47^.

For the transition between swirls and swarms, we use the deviation angle Δ as the control parameter and measure susceptibility *χ* and rotational order $\left\langle {\left| {O_{\mathrm{R}}} \right|} \right\rangle$ of the group. The finite-size scaling is then given by ${\tilde{\mathrm{\Delta }}} = \frac{{{\mathrm{\Delta }} \, - \, {\mathrm{\Delta }}_c}}{{{\mathrm{\Delta }}_c}} \cdot N^{1/\nu }$, $\tilde \chi = \chi \cdot N^{ - \gamma /\nu }$, $\widetilde {\left\langle {\left| {O_{\mathrm{R}}} \right|} \right\rangle } = \left\langle {\left| {O_{\mathrm{R}}} \right|} \right\rangle \cdot N^{\beta /\nu }$, with the critical deviation angle Δ_*c*_ and the critical exponents *ν*, *γ*, *β* of correlation length, susceptibility and order parameter, respectively.

The values of Δ_*c*_, *ν* and *γ* are determined by fitting all three parameters simultaneously using *χ* vs. Δ curves obtained from simulations with *N* = [100, 150, 250, 500]. The best data collapse is achieved for *ν* = 3.87, *γ* = 2.24 and Δ_*c*_ = 56.15°. *β* = 0.66 is fitted independently using the corresponding $\left\langle {\left| {O_{\mathrm{R}}} \right|} \right\rangle$ vs. Δ curves, and keeping the previously determined values of *ν* and Δ_*c*_ fixed. We want to mention that the simulation data for *N* = 50 particles in Fig. 4c–f were not considered for the fitting procedure because at such small system sizes, only partial data collapse can be achieved.

### Realisation of virtual obstacles

**Fig. 7:**
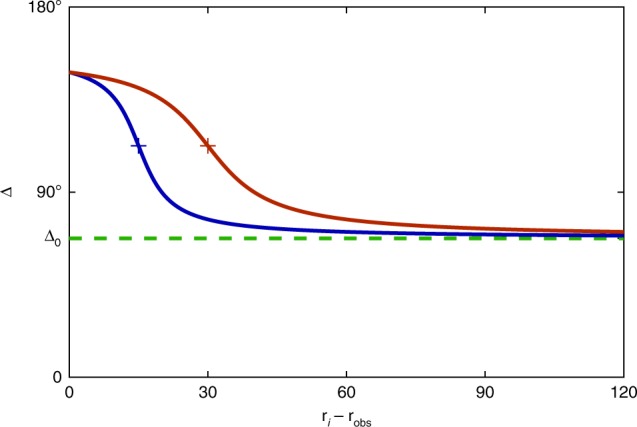
Interaction rule for particles encountering an obstacle. Deviation angle Δ in dependence of the distance of an AP to the obstacle centre for obstacles with 15- μm (blue) and 30-μm (red) radius. Markers indicate the obstacle radii corresponding to the data shown in Fig. 4d.

In our experiments, we introduce virtual obstacles by defining a zone of enhanced angular deviation. This is realised by making the deviation angle Δ_*i*_ now depending on the distance between the position **r**_*i*_ of particle *i* and the centre of the obstacle **r**_obs_. Δ_*i*_ is given by ${\mathrm{\Delta }}_i\left( {\left| {{\mathbf{r}}_i - {\mathbf{r}}_{{\mathrm{obs}}}} \right|} \right) = {\mathrm{\Delta }}_0 + {\mathrm{\Delta }}_{{\mathrm{obs}}}\left( {\frac{1}{2} - \frac{1}{\pi }\arctan \left( {\frac{{\left| {{\mathbf{r}}_i \, - \, {\mathbf{r}}_{{\mathrm{obs}}}} \right| \, - \, R_{{\mathrm{obs}}}}}{{w_{{\mathrm{obs}}}}}} \right)} \right)$, where Δ_0_ is the deviation angle without an obstacle, Δ_obs_ the amplitude of the additional deviation, *R*_obs_ the radius and *w*_obs_ the width of the obstacle edge, respectively (Fig. 7). To avoid spontaneous rotation reversals during the measurements, the sign of the deviation angle was fixed to allow only counterclockwise motion. Translation of the obstacle can be achieved by defining a time-dependent position of the obstacle **r**_obs_(*t*). In Fig. 5j, **r**_obs_(*t*) moves with a constant velocity on a circular trajectory.

### Numerical simulations

To simulate AP trajectories, we numerically integrated the overdamped coupled two-dimensional equations of motion for the position **r** and orientation ${\hat{\mathbf{u}}}$ of each particle (note that **r** and ${\hat{\mathbf{u}}}$ are complex values corresponding to the *x* and *y* components) ${\mathbf{r}}\left( {t + \tau } \right) = {\mathbf{r}}\left( t \right) + \tau v{\hat{\mathbf{u}}} + {\mathbf{\zeta }}_{\mathrm{T}} \cdot \sqrt \tau$ and ${\hat{\mathbf{u}}}\left( {t} + {\tau} \right) = {\hat{\mathbf{u}}}\left( {t} \right) \cdot \exp \left( {i}\left( {\tau} \frac{{\Gamma} _{\mathrm{max}}}{k_{\mathrm{B}}{T}}{D_{\mathrm{R}}}{\sin} \left( {\gamma} \left( {t} \right) \right) + {\zeta _{\mathrm{R}}} \cdot {\sqrt {\tau}} \right) \right)$ with *γ*(*t*) the angle between orientation and steering direction (Fig. 6a). ***ζ***_T_ and *ζ*_R_ correspond to random forces with zero mean and variance $\left\langle {\mathbf{\zeta}}_{\mathrm{T}}^i\left( t \right){\mathbf{\zeta}}_{\mathrm{T}}^j\left( {t^{\prime}} \right) \right\rangle = 2D_{\mathrm{T}}\delta _{ij}\delta \left( {t - t^{\prime}} \right)$ and $\left\langle \zeta _{\mathrm{R}}\left( t \right)\zeta _{\mathrm{R}}\left( {t^{\prime}} \right) \right\rangle = 2D_{\mathrm{R}}\delta \left( {t - t^{\prime}} \right)$, respectively. The parameters are set, except otherwise stated, to the experimental values: velocity *ν* = 0.5 μm s^−1^, torque Γ_max_ = 25 *k*_B_*T* and translational and rotational diffusion constants *D*_T_ = 0.014 μm^2^ s^−1^ and *D*_R_ = 0.0028 s^−1^. For the simulation time step, we choose *τ* = 0.2 s as a further reduction does not lead to changes in the simulation results.

To model hard sphere-like interactions between APs, after each integration step, we check for overlapping particles, i.e., distances < *σ*. These overlaps are recursively treated by shifting overlapping pairs of particles away from their common centre of mass until overlaps vanish.

## Supplementary information

Supplementary Information

Description of Additional Supplementary Files

Supplementary Movie 1

Supplementary Movie 2

Supplementary Movie 3

## Data Availability

The data that support the plots within this paper and other findings of this study are available from the corresponding author upon reasonable request.
