# Supplementary material for: Formation of stable and responsive collective states in suspensions of active colloids
Source: Nat Commun. 2020 May 21;11:2547. doi: 10.1038/s41467-020-16161-4 (PMC7242396; doi:10.1038/s41467-020-16161-4)
Supplement: Supplementary file 1 — Supplementary Information [file 41467_2020_16161_MOESM1_ESM.pdf]

Supplementary Information for

Formation of stable and responsive collective states in suspensions of active colloids

Bäuerle et. al

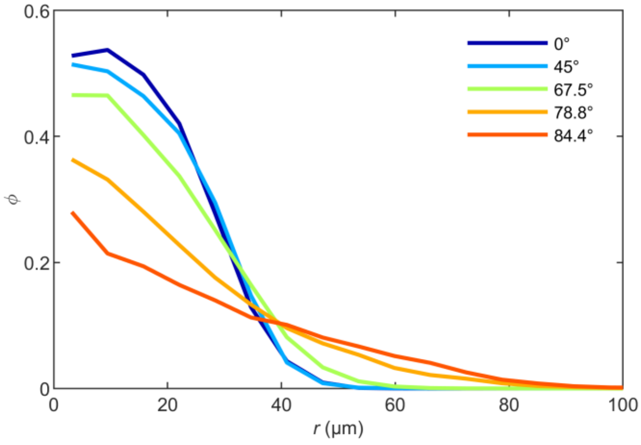

Supplementary Figure 1 | Radial area fraction profiles for different deviation angles Δ

Radial area fraction profile of swirls composed of 50 particles for different values of the deviation angle Δ and $\alpha = 360°$, $R_{\mathrm{p}} = \infty$.

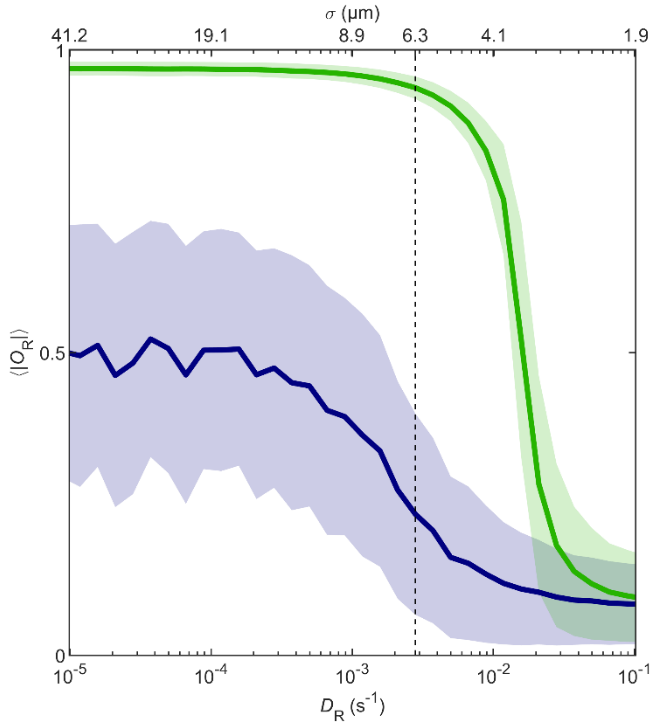

Supplementary Figure 2 | Influence of rotational diffusion on swirl formation

Influence of the rotational diffusion coefficient D_R on the rotational order parameter O_R. The data compares numerical simulations of the interaction rule in this work (green, see Fig.1a-c) and a typical zonal model [1] (blue). Curves are averaged over 20 simulations each with a length of 3 hours, error bands correspond to the standard deviation. The model dependent parameters are $R_\mathrm{r} = 8\ \mu\mathrm{m}$ (corresponding to a minimum clearance of ≈ 0.25), $R_\mathrm{o} = 25\ \mu\mathrm{m}$, $R_\mathrm{a} = \infty$, $\Delta = 67.5°$, $\alpha = 270°$ (see Fig. 1a-c) and $r_\mathrm{r} = 8\ \mu\mathrm{m}$, $r_\mathrm{o} = \Delta r_0 + r_\mathrm{r} = 25\ \mu\mathrm{m}$, $r_\mathrm{a} = \Delta r_\mathrm{a} + r_\mathrm{o} = \infty$, $\alpha = 270°$ (parameters defined as in [1]), respectively.

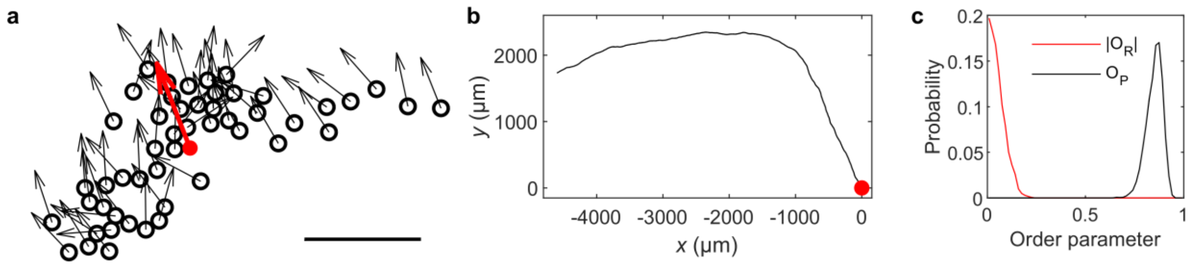

Supplementary Figure 3 | Creation of stable flocks

a Simulated snapshot of a group that is in a stable flocking state due to R_o being larger than the size of the group. Black arrows show the swimming direction of each particle and centre-of-mass is shown in red. Scale bar is $50\ \mu\mathrm{m}$. **b** Flock's centre-of-mass motion corresponding to a duration of 4 hours. **c** Histogram of the polarisation O_P and the rotational order $|O_\mathrm{R}|$ of the group. Simulation parameters are $N = 50$ particles, $\alpha = 360°, \Delta = 67.5°,\ R_\mathrm{o} = R_\mathrm{a} = \infty$.

Supplementary References

1. Couzin, I. D., Krause, J., James, R., Ruxton, G. D. & Franks, N. R. Collective memory and spatial sorting in animal groups. *J. Theor. Biol.* **218**, 1–11 (2002).
