# Supplementary material for: Formation of stable and responsive collective states in suspensions of active colloids
Source: Nat Commun. 2020 May 21;11:2547. doi: 10.1038/s41467-020-16161-4 (PMC7242396; doi:10.1038/s41467-020-16161-4)
Supplement: Supplementary file 2 — Description of Additional Supplementary Files [file 41467_2020_16161_MOESM2_ESM.pdf]

Description of Additional Supplementary Files

Supplementary Movie 1 | Cohesive swirl of APs

Swirling group of 50 APs with parameters $R_\mathrm{a} = 60\ \mu\mathrm{m}$, $\alpha = 315°$ and $\Delta = 67.5°$. The group shows counter-clockwise rotation. Fading trajectories with a length of $150\,s$ indicate the motion of the individual APs. The video is accelerated by a factor of 120 and the scale bar is $20\ \mu\mathrm{m}$.

Supplementary Movie 2 | Fragmenting group of APs

Group of 50 APs with parameters $R_\mathrm{a} = 150\ \mu\mathrm{m}$, $\alpha = 225°$ and $\Delta = 67.5°$. Due to restricted vision, the group is not cohesive but fragments. Fading trajectories with a length of $150\,s$ indicate the motion of the individual APs. The video is accelerated by a factor of 120 and the scale bar is $20\ \mu\mathrm{m}$.

Supplementary Movie 3 | Translating obstacle within a swirl of APs

Swirling group of APs following a virtual obstacle (orange) which is translating on a circular trajectory with $30\ \mu\mathrm{m}$ radius. Experimental parameters are $R_\mathrm{a} = \infty$, $\alpha = 360°$, $\Delta_0 = 67.5°$, $\Delta_\mathrm{obs} = 180°$, $R_\mathrm{obs} = 15\ \mu\mathrm{m}$ and $w_\mathrm{obs} = 5\ \mu\mathrm{m}$. Fading trajectories with a length of $150\,s$ indicate the motion of the individual APs. The video is accelerated by a factor of 240 and the scale bar is $20\ \mu\mathrm{m}$.
